# Tissue Engineering the Pinna: Comparison and Characterization of Human Decellularized Auricular Biological Scaffolds

**DOI:** 10.1021/acsabm.1c00766

**Published:** 2021-08-31

**Authors:** Zaid Al-Qurayshi, Emad I. Wafa, Monica K. Rossi Meyer, Scott Owen, Aliasger K. Salem

**Affiliations:** † Department of Otolaryngology − Head & Neck Surgery, University of Iowa Hospitals and Clinics, Iowa City, Iowa 52242, United States; ‡ Department of Pharmaceutical Sciences and Experimental Therapeutics, College of Pharmacy, University of Iowa, Iowa City, Iowa 52242, United States; § Holden Comprehensive Cancer Center, University of Iowa, Iowa City, Iowa 52242, United States

**Keywords:** tissue engineering, regenerative medicine, auricle, pinna, decellularization, biological scaffold, plastic surgery

## Abstract

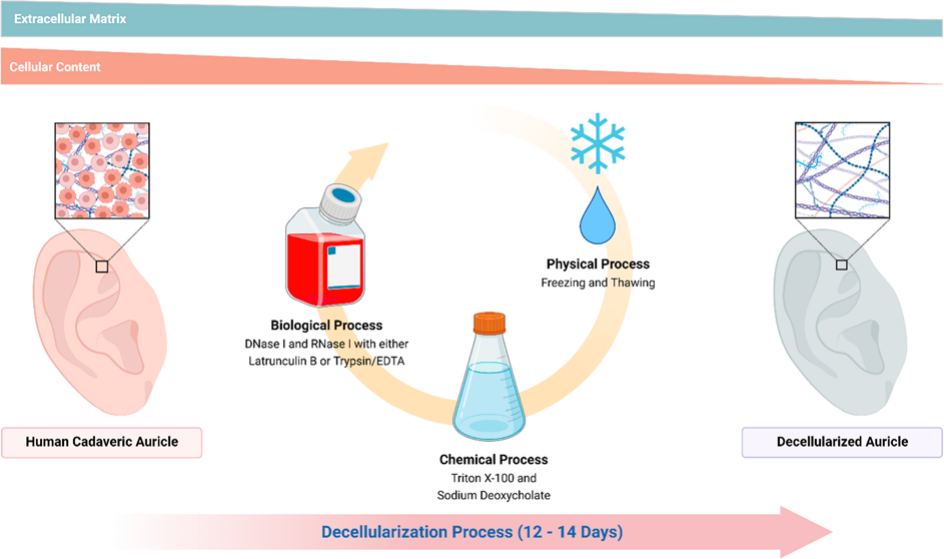

Decellularization is one of the promising techniques in tissue engineering used to create a biological scaffold for subsequent repopulation with the patient’s own cells. This study aims to compare two different decellularization protocols to optimize the process of auricle decellularization by assessing and characterizing the decellularization effects on human auricular cartilage. Herein, 12 pairs (8 females, 4 males) of freshly frozen adult human cadaveric auricles were de-epithelialized and defatted leaving only the cartilaginous framework. An auricle from each pair was randomly assigned to either protocol A (latrunculin B-based decellularization) or protocol B (trypsin-based decellularization). Gross examination of the generated scaffolds demonstrated preservation of the auricles’ contours and a change in color from pinkish-white to yellowish-white. Hematoxylin and eosin staining demonstrated empty cartilaginous lacunae in both study groups, which confirms the depletion of cells. However, there was greater preservation of the extracellular matrix in auricles decellularized with protocol A as compared to protocol B. Comparing protocol A to protocol B, Masson’s trichrome and Safranin-O stains also demonstrated noticeable preservation of collagen and proteoglycans, respectively. Additionally, scanning electron micrographs demonstrated preservation of the cartilaginous microtopography in both study groups. Biomechanical testing demonstrated a substantial decrease in Young’s modulus after decellularization using protocol B (1.3 MPa), albeit not significant (*P*-value > 0.05) when compared to Young’s modulus prior to decellularization (2.6 MPa) or after decellularization with protocol A (2.7 MPa). A DNA quantification assay demonstrated a significant drop (*P*-value < 0.05) in the DNA content after decellularization with protocol A (111.0 ng/mg) and protocol B (127.6 ng/mg) in comparison to before decellularization (865.3 ng/mg). Overall, this study demonstrated effective decellularization of human auricular cartilage, and it is concluded that protocol A provided greater preservation of the extracellular matrix and biomechanical characteristics. These findings warrant proceeding with the assessment of inflammation and cell migration in a decellularized scaffold using an animal model.

## 1 Introduction

**Figure 1:**
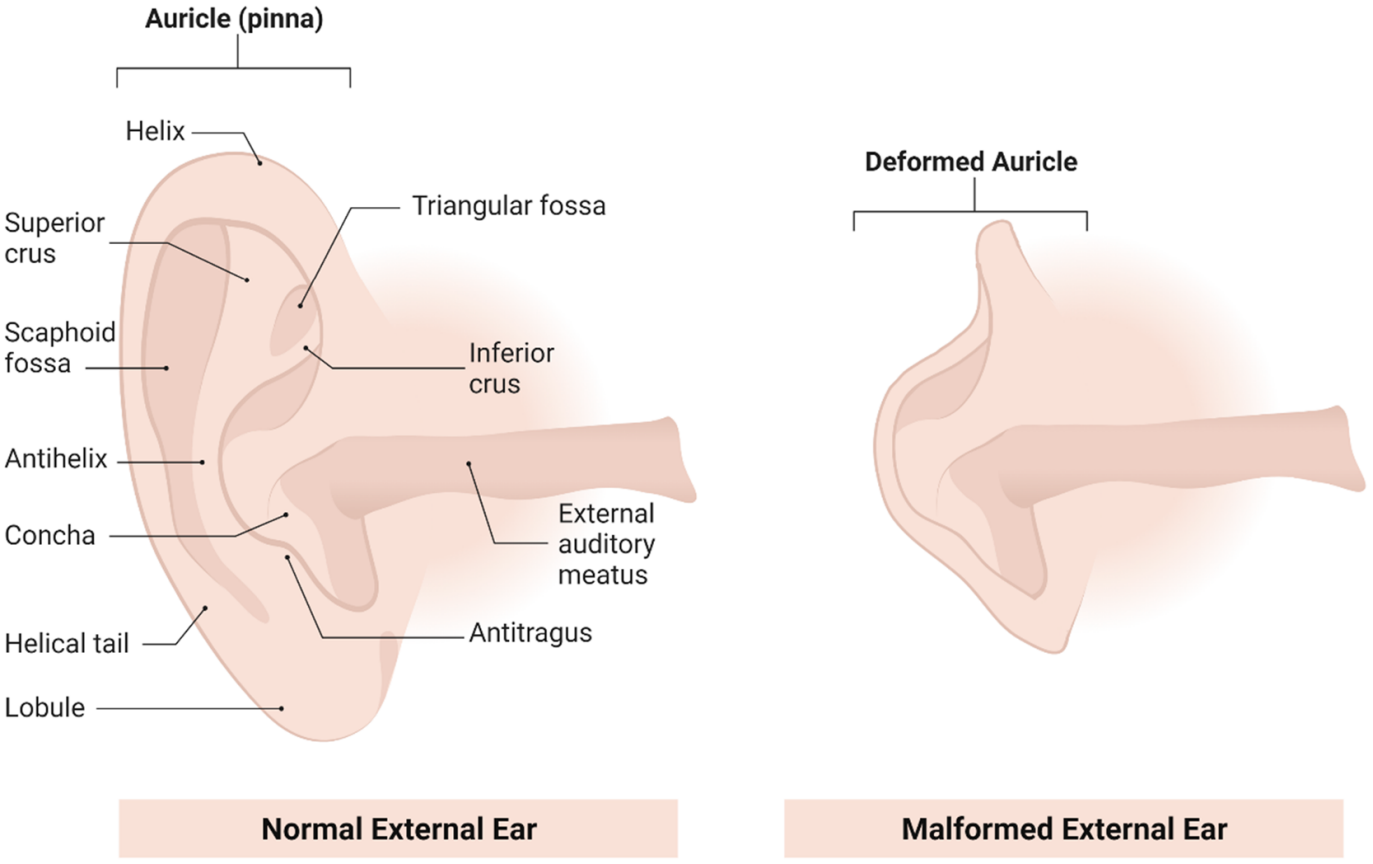
Schematic representation of the external ear anatomy showing the structural differences between the normal healthy auricle and the deformed auricle (microtia).

Microtia is a developmental anomaly of the auricle that results in a small or deformed pinna (Figure 1).^1,2^ Its overall incidence is estimated to be 1–3 per 10 000 births. However, variation among populations exists where a higher incidence has been observed in Asians and certain Native American populations.^1,3−6^ The incidence is higher in male patients with a male:female ratio of 2.5:1, and the right side is more commonly involved than the left with 80% of patients having only one side involved.^1^ Congenital deformity of the external ear or malformation due to trauma or burns can cause negative psychological and social effects.^7,8^ Additionally, microtia is associated with other ramifications such as hearing loss and ear infection. Although surgical reconstruction of microtia is recommended in most cases, it is technically challenging and difficult to achieve a desirable aesthetic outcome. The reconstruction is usually performed with autologous rib-costal cartilage fashioned to mimic the contours of the pinna in a single or a staged procedure.^1,9,10^ However, due to the differences in the mechanical properties and the degree of flexibility between the costal cartilage (consisting of an amorphous gelatinous matrix with a high density of type II collagen fibers) and the auricular elastic cartilage (containing high-density elastic fibers), costal cartilage grafts are an imperfect framework for reconstructive ear surgery.^11^ In other words, there are difficulties in mimicking the native, unique extracellular matrix of the auricle with autologous cartilage grafts.

Recent advances in regenerative medicine and tissue engineering are making the leap from the laboratory bench to clinical application.^12^ Furthermore, tissue engineering has been increasingly recognized for its potential future application in medicine to overcome shortages of donated organs and for its technical versatility in fabricating the desired tissue.^13,14^ In addition to circumventing organ donation, another appealing advantage of tissue engineering is obviating the need for immunosuppressing the recipient of a tissue-engineered organ.^12^ Over the past decade, tissue engineering and 3D printing have emerged and been tested for their utility in auricular reconstruction.^1^ Synthetic 3D printed auricular frameworks have been investigated as a substitute for harvesting autologous cartilage, with promising results.^15,16^ However, synthetic scaffolds have been observed to be complicated by extrusion and fracture.^1^

An alternative approach to synthetic scaffolds is to utilize decellularized biological scaffolds of the auricle with the advantage that they retain their native extracellular matrix. Because of this, biological scaffolds obtained from tissue decellularization are increasingly being recognized in multiple fields of tissue engineering as a preferred alternative to synthetic scaffolds.^17−21^ The recent advances in the field of tissue engineering have provided the opportunity to engineer functional and biological auricular scaffolds using cadaveric auricles. The advantages of the biological scaffold are attributed to the preservation of microstructure that cannot be reproduced with synthetic material and the residual effect of native growth factors within a construct.^22^ The objective of this study was to examine and compare the effectiveness of two previously described decellularization protocols to generate auricular cartilaginous scaffolds that could ultimately be used in reconstructing the auricle in patients with microtia. Our study consisted of applying two different decellularizing protocols to achieve optimum decellularization of the human auricle. Protocol A involved latrunculin B-based decellularization, while protocol B used a trypsin-based decellularization process.

## 2 Experimental Section

### 2.1 Ethics

This study was approved by the University of Iowa Human Subjects Office (HSO) and was in compliance with the University of Iowa Institutional Review Boards (IRBs) for research involving biospecimens from deceased individuals.

### 2.2 Specimens

Twelve pairs of adult cadaveric auricles were obtained through the University of Iowa Deeded Body Program. The specimens were freshly frozen and had not been treated with any chemicals. Samples from each specimen were collected before and after decellularization using punch biopsy (6 mm) taken from the triangular fossa and adjacent area. The samples were divided among the characterization tests performed as explained below. Each specimen acted as its own control for the comparison of changes before and after decellularization.

### 2.3 Study Design

The specimens were randomly assigned into two groups using a Microsoft Excel random function. The randomization process was designed so that each group received one of the auricle pair from each donor, and each group in total received six right-sided auricles and six left-sided auricles. Each group was decellularized following previously published and modified protocols as discussed below.

### 2.4 Decellularization Process Using Protocol A

Protocol A was adapted with modification from a previously described protocol developed by Ansari et al.^7^ Ansari et al. demonstrated successful decellularization of the larynx. The protocol involves biological, chemical, and physical decellularization methods. All decellularization steps were performed while the specimens were kept under constant agitation (100 rpm) using a shaker, and all solutions contained 1% penicillin/streptomycin (Gibco, Life Technologies Corp.). Between every decellularization step, the auricles were washed for 15 min twice with Nanopure autoclaved water. The decellularization of each auricle took 12 days. In detail, the decellularization process was as follows: first, the auricles were thawed at room temperature for approximately 1 h. Next, the specimens were placed in Dulbecco’s Modified Eagle Medium (DMEM) (Gibco, Life Technologies Corp.) and latrunculin B (Tocris Bioscience) 50 nmol/L solution for 2 h at 37 °C. Afterward, the auricles were washed and placed in 0.6 mol/L KCl (Fisher Scientific) solution for 2 h at room temperature, followed by another washing step, then placed in 1 mol/L KI (Fisher Scientific) for 2 h at room temperature. Subsequently, the auricles were left to wash overnight at room temperature. On the second day, the immersion in KCl and KI step was repeated, followed by incubation in a solution containing 2 units/mL DNase I (New England BioLabs Inc.) in water for 2 h at 37 °C. The auricles were washed and frozen overnight. The following day (day 3), the auricles were left to thaw at room temperature for 24 h. On the fourth day, the auricles were incubated in 0.25% Triton X-100 and 0.25% w/v sodium deoxycholate in phosphate-buffered saline (PBS) (Sigma-Aldrich, St. Louis, MO) for 24 h at 37 °C. For the following 2 days, the auricles were left for 24 h at 4 °C. On the seventh day, the auricles were incubated in 2 units/mL DNase I and 0.1 g/L RNase I in distilled water for 24 h at 37 °C. On the following day, the auricles were washed for 24 h at 4 °C. The incubation in the DNase I and RNase I solution was repeated. Subsequently, over the following 3 days, the auricles were left to wash for 24 h at 4 °C (i.e., three times of the 24-h wash cycle). This concluded the decellularization process, and the specimens were frozen at −20 °C.

### 2.5 Decellularization Process Using Protocol B

Protocol B was adapted with modification from a study published by Rahman et al.^22^ In their study, they compared three protocols of auricle decellularization. They demonstrated that the addition of trypsin resulted in a significant reduction in DNA content and cell depletion.^22^ In this study, all steps were performed under constant agitation of 100 rpm, and all solutions used contained 1% penicillin/streptomycin. First, the auricles were left to thaw at room temperature for approximately 1 h; then they were wet-frozen in Nanopure autoclaved water overnight. The next day, the auricles were left to thaw at room temperature and then washed in water overnight. On the third day, the auricles were placed in 0.25% trypsin/EDTA (Gibco, Life Technologies Corp.) for 2 h at 37 °C. This was followed by two 15 min washing steps in water. The trypsinization process and washing steps were then repeated, followed by placing the auricles in 2 units/mL DNase I in water for 2 h. The auricles were left to wash overnight at 4 °C. The fourth and fifth days repeated the same steps performed on the third day. On the sixth day, the auricles were incubated in 0.25% Triton X-100 and 0.25% w/v sodium deoxycholate in PBS for 24 h at 37 °C. For the following 2 days, the auricles were left for 24 h at 4 °C. On the ninth day, the auricles were incubated in 2 units/mL DNase I and 0.1 g/L RNase in distilled water for 24 h at 37 °C. On the following day, the auricles were washed for 24 h at 4 °C. The incubation in the DNase I and RNase I solution was repeated. Subsequently, over the following 3 days, the auricles were left to wash for 24 h at 4 °C. This concluded the decellularization process; the specimens were then frozen at −20 °C.

### 2.6 Histological Architecture

Specimens obtained using punch biopsy (6 mm) were taken from the triangular fossa and adjacent area before and after decellularization. The specimens were then exposed to different staining methods to evaluate the changes in the microanatomy of the auricles after being decellularized using the two different protocols. All stained samples were visualized using a CKX41 inverted microscope equipped with a DP70 digital camera system (Olympus).

#### 2.6.1 Hematoxylin and Eosin (H&E)

This stain was primarily used for cellular detection and the overall distribution of cells in the cartilage before and after decellularization. Specimens were initially fixed in 10% neutral buffered formalin solution (Research Products International, Mount Prospect, IL) for approximately 24 h. This was followed by embedding the specimens in paraffin blocks and slicing the paraffin blocks into thin sections. Next, the tissue sections were mounted onto microscope slides and subjected to dehydration and rehydration steps for subsequent staining and analysis. Rehydration involved 3 × 10 min xylene (deparaffinization), 3 × 1 min 100% alcohol, 1 × 1 min 95% alcohol, 1 × 1 min 70% alcohol, followed by a rinse in distilled water. Dehydration was the reverse timing process. Slides were then stained with the basic nuclear stain (Harris hematoxylin, Leica Biosystems, Buffalo Grove, IL) and acidic stain (eosin, Leica Biosystems), respectively. The staining time was approximately 3 min for each stain. Samples were thoroughly rinsed with distilled water between and after the staining steps to remove excess or nonspecific background stain.

#### 2.6.2 Masson’s Trichrome

This staining technique was used to visualize collagen fibers. Paraffin-embedded specimens were processed onto microscope slides as described in the above section. The staining process involved a combination of Weigert’s hematoxylin, 2.5% aqueous phosphomolybdic-phosphotungstic acid, Bouin’s solution, working Biebrich Scarlet-acid fuchsin 1% aqueous solution, and 2.5% aniline blue solution (Sigma-Aldrich), with more in depth details having been previously reported.^23^

#### 2.6.3 Safranin-O

This cationic stain has been commonly used to evaluate the proteoglycan content in cartilage. Similar to the prestaining process used for H&E staining (above), specimens were mounted onto microscope slides. The staining methodology involved using both 0.1% Safranin-O (2 min) and 0.1% Fast Green (5 min) stains (Sigma-Aldrich), with more in depth details having been previously reported.^23^ Finally, slides were imaged using a light microscope.

### 2.7 Surface Topography

Electron scanning microscopy was used to examine the microstructure and surface topography of the cartilage as demonstrated by Rahman et al.^22^ Briefly, auricle sections were coated with a mixture of gold and palladium using an argon beam K550 sputter coater (Emitech Ltd.). Scanning electron micrographs (SEM) were then captured using a Hitachi S-4800 scanning electron microscope (Hitachi High-Technologies).

### 2.8 Mechanical Properties (Young’s Modulus)

Digital Vernier calipers were used to measure the thickness of each sample. Cartilage mechanical properties were assessed using a 1 kg load cell on the Insight Electromechanical Testing System (MTS). The cartilage was immersed in PBS, while a 2 mm diameter hemispherical indenter was brought into contact with the cartilage surface. Once contact was established, the cartilage was loaded to 20% strain at 1 mm/s, and then allowed to relax for 10 min while the compression was held at 20% strain. In this experiment, data were recorded at 1 kHz for the initial loading phase and at 500 Hz for relaxation. The compressive Young’s modulus was calculated as the linear fit of the stress/strain curve over the range of 10–15% strain, with Hertzian contact assumed for the spherical indenter. The final relaxed stress at the end of the test was also reported, as well as the ending stress–relaxation rate (MPa/s) from the last 100 s of data.

### 2.9 DNA Quantification

Quantification of DNA was assessed for each specimen using DNeasy (Qiagen). The assay was performed following the manufacturer’s protocol. In summary, a full-thickness piece of cartilage was obtained from three pairs (a total of six pinnas, three from each study group). The auricle pairs were randomly selected from the total pairs included in the study. The weight of each cartilage specimen was recorded. The specimen was lysed used proteinase K while being incubated at 56 °C. This was followed by multiple steps of centrifugation using a manufacturer-provided buffer solution aimed at isolating the DNA content using manufacturer-provided spin tubes. The DNA content in the isolates was then estimated using spectrophotometry. Absorbance readings were measured at 260 nm using a SpectraMax Plus 384 microplate reader (Molecular Devices, San Jose, CA).

### 2.10 Sulfated Glycosaminoglycan (sGAG) Quantification

In this study, sGAG content in each specimen was quantified using the Blyscan sGAG Assay (Biocolor). The assay was performed following the manufacturer’s protocol. In summary, a full-thickness piece of cartilage was obtained from three pairs (a total of six pinnas, three from each study group). The auricle pairs were randomly selected from the total pairs included in the study. The weight of each cartilage specimen was recorded. The sGAG was extracted from the tissue using a Papain extraction reagent. A dye was then added to form a complex with sGAG. The dye-bound sGAG was precipitated and drained. Subsequently, a dissociation agent was added to unbind the sGAG from the dye. The recovered sGAG content was measured at 656 nm using spectrophotometry (SpectraMax Plus 384 microplate reader) and estimated using a standardized curve.

### 2.11 Statistical Analysis

In this study, collected data were first analyzed by one-way ANOVA using an F-test. Subsequently, a Tukey’s multiple comparison test was performed to compare all pairs of groups (i.e., comparing means of the parameter of interest among the study groups). Statistical analysis was carried out using GraphPad-Prism 8 (GraphPad Software), and differences were considered statistically significant when *p* < 0.05.

## 3 Results

### 3.1 Gross-Structural Characteristics

**Figure 2:**
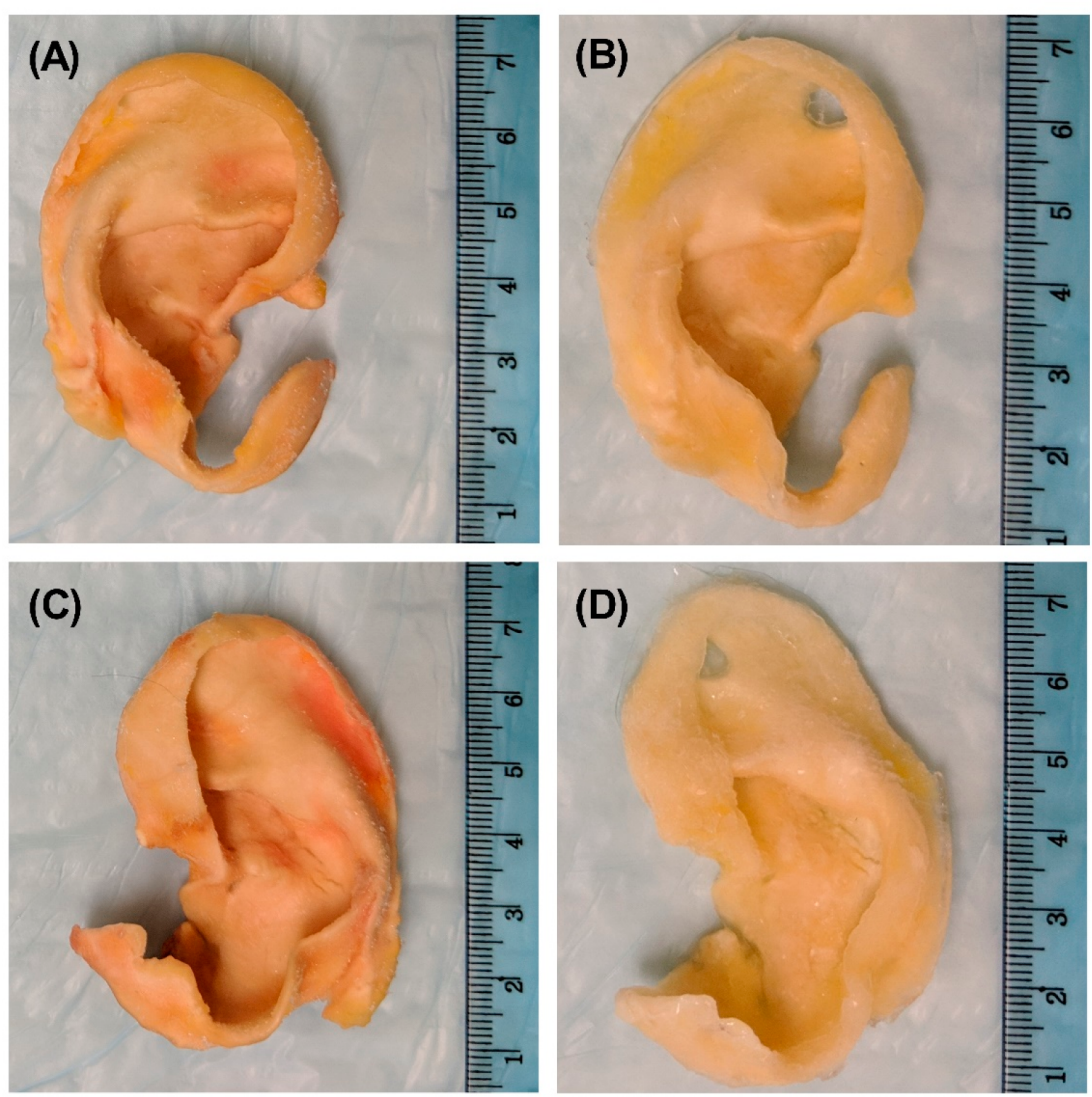
Gross structural characteristics of auricles before and after decellularization. Images showing one of the auricles (A) before (native) and (B) after decellularization using protocol A; and one of the auricles (C) before and (D) after decellularization using protocol B.

In this work, 12 pairs of adult cadaveric auricles were used to evaluate the impact of tissue decellularization using two different protocols. The donors’ information including age and gender as well as the group allocation for each individual auricle are summarized in Table 1. Both decellularization protocols (A and B) were applied as planned where protocol A by design took 12 days and protocol B took 14 days to conclude for each auricle. Gross examination of the generated scaffolds after decellularization demonstrated preservation of the auricles’ contours and a change in color from light pink to light yellow (Figure 2). Overall, the decellularization process did not alter the gross appearance of the auricles. In addition, evaluation of auricle tissue dimensions revealed that neither decellularization process resulted in any discernible changes.

**Table 1:** Characteristics of the Specimens’ Donors and Their Study Group Allocation

donor	gender	age (year)	protocol A	protocol B
1	F	88	right	left
2	F	100	left	right
3	F	63	left	right
4	F	71	right	left
5	M	92	left	right
6	F	98	right	left
7	M	82	left	right
8	F	80	right	left
9	F	93	left	right
10	F	82	left	right
11	M	64	right	left
12	M	87	right	left

### 3.2 Histological and Microstructural Characteristics

**Figure 3:**
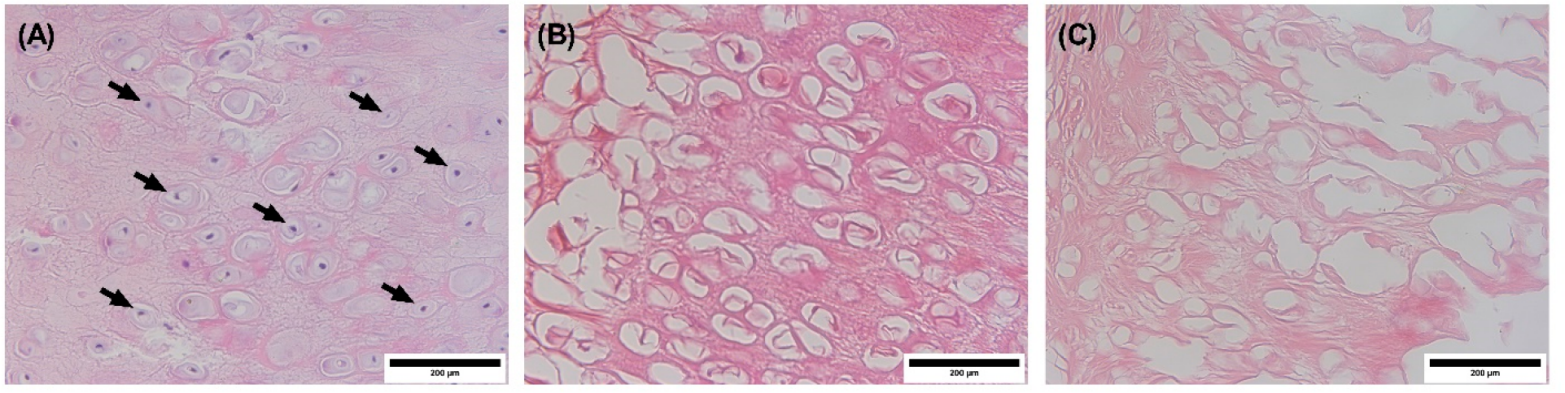
Images of H&E stained sections visualized by a light microscope. (A) Auricle before decellularization (native auricle, arrows point to nuclei); (B) and (C) represent the decellularized auricles using protocol A and protocol B, respectively. The nuclei are stained blue (hematoxylin), while the extracellular matrix and cytoplasm are stained with varying degrees of pink (eosin). Images were captured at 40× objective magnification. Scale bar = 200 μm.

Auricular specimens were stained with different reagents and examined under a light microscope to assess any changes in the microanatomy of the decellularized scaffolds. The histological H&E stained section of the punch biopsy from the auricle prior to decellularization showed normal tissue structure with visible cell boundaries and nuclei (Figure 3A). In addition, examination of H&E stained decellularized samples using light microscopy revealed empty cartilaginous lacunae in both study groups, suggesting cell depletion in these cavities (Figure 3B and C). However, the extracellular matrix was more preserved in the specimens decellularized using protocol A as compared to the specimens decellularized with protocol B.

**Figure 4:**
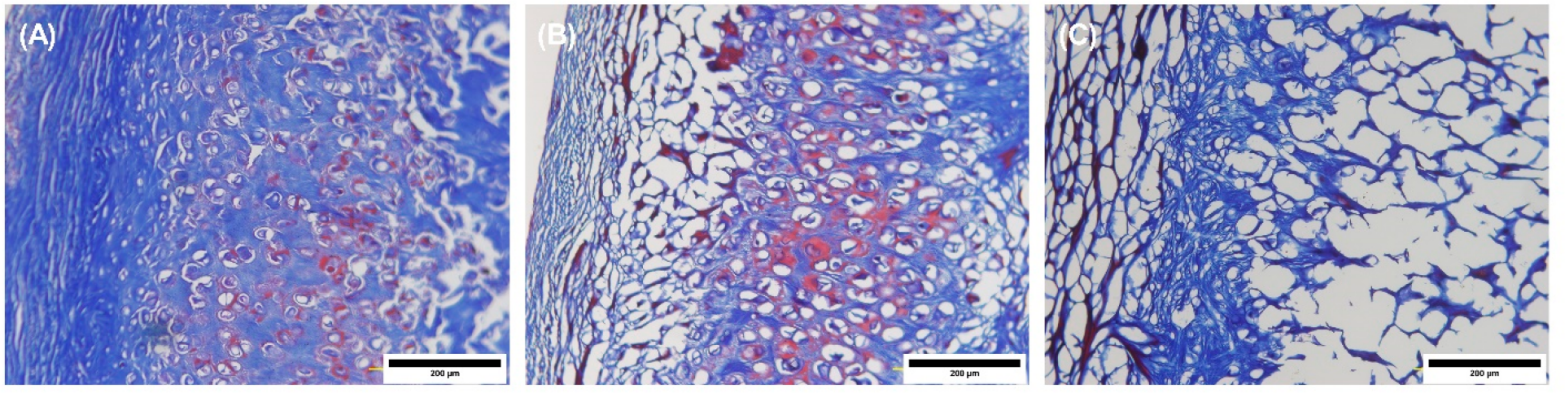
Images of Masson’s trichrome stained sections of punch biopsies from auricles. (A) Auricle before decellularization (native auricle); (B) and (C) represent the decellularized auricles using protocol A and protocol B, respectively. The blue-stained areas indicate the presence of collagen. Images were captured at 20× objective magnification. Scale bar = 200 μm.

Staining the specimens with Masson’s trichrome stain indicated that the auricle had an abundant amount of collagen prior to decellularization (Figure 4A). Upon decellularization using protocol A, the auricle exhibited a marginal reduction in the amount of collagen (Figure 4B and C). Also, it was observed that the auricle decellularized with protocol B had less collagen than the other auricles as evidenced by a lower color (blue) intensity.

**Figure 5:**
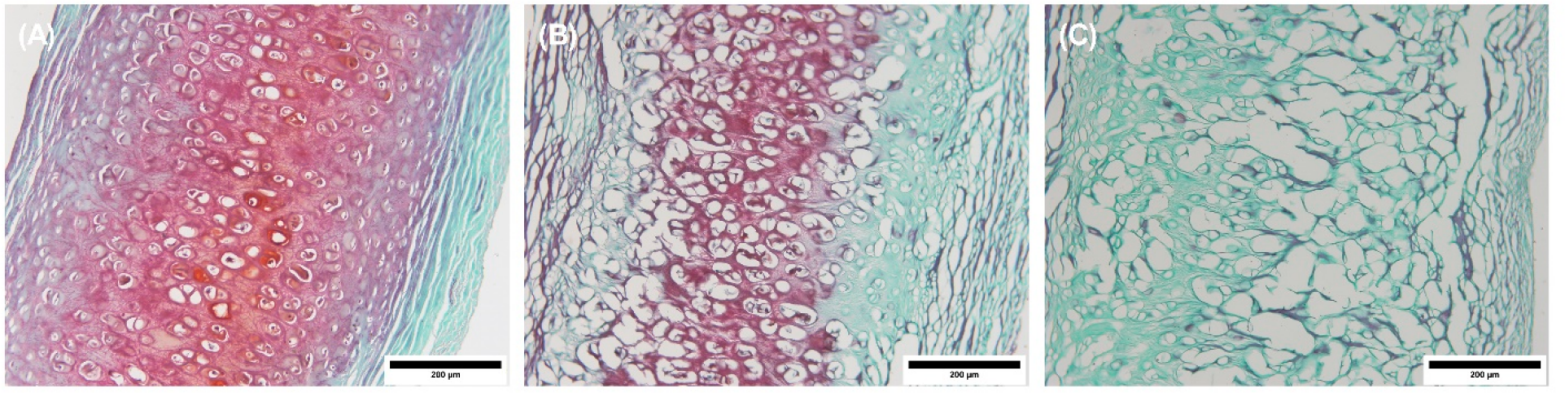
Images of Safranin-O stained sections of punch biopsies from auricles. (A) The auricle before decellularization (native auricle); (B) and (C) represent the decellularized auricles using protocol A and protocol B, respectively. The red-stained areas indicate the presence of proteoglycan. Images were captured at 20× objective magnification. Scale bar = 200 μm.

The proteoglycan content, as indicated by the red color intensity of the extracellular matrix (stained with Safranin-O), was apparent in the image of the auricle prior to decellularization (Figure 5A). However, a decreased level of staining (i.e., less proteoglycan content) was observed in the Safranin-O stained sections of the decellularized auricle using protocol A (Figure 5B). Further reduction in the proteoglycan content was noticed in the auricle sections decellularized with protocol B (Figure 5C).

### 3.3 Surface Topography

**Figure 6:**
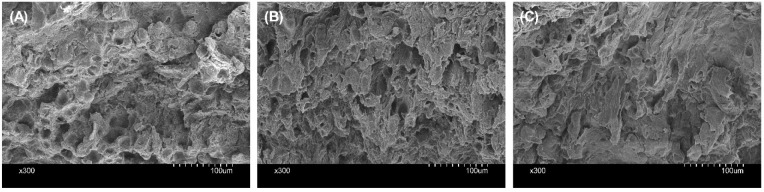
Scanning electron micrographs illustrating histological features of the human auricle. (A) The auricle before decellularization (native auricle); (B) and (C) represent the decellularized auricles using protocol A and protocol B, respectively. Images were captured at 300× magnification. Scale bar = 100 μm.

Scanning electron microscopy was utilized to examine the structure of the cartilage. SEM of auricles decellularized using protocol A demonstrated the preservation of the cartilaginous microtopography when compared to auricles prior to decellularization (i.e., native auricle) (Figure 6A and B). However, auricles decellularized with protocol B exhibited marginal loss of the cartilage structure (Figure 6C).

### 3.4 Mechanical Properties and DNA/sGAG Content

**Figure 7:**
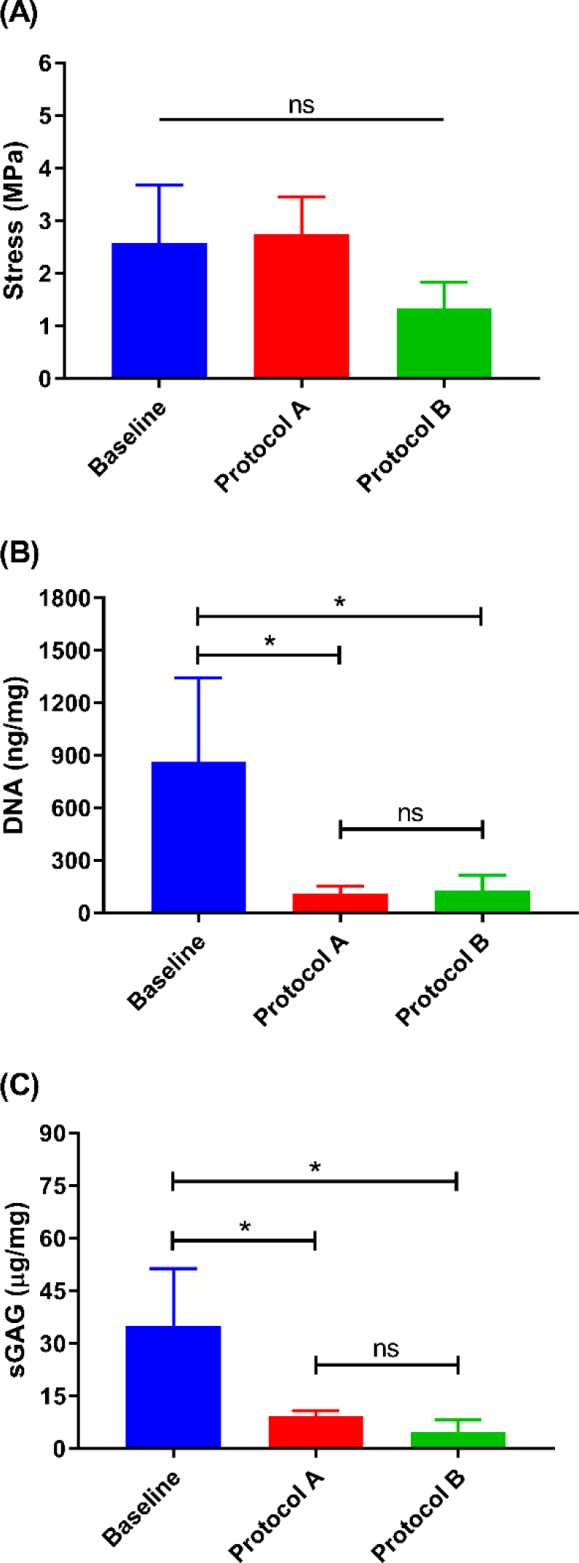
Characteristics of the human auricles before and after two different decellularization methodologies. (A) Indentation test to evaluate the stress. (B) DNA content. (C) sGAG content. Data were plotted as mean ± standard deviation. ns, not statistically significant (i.e., *p* > 0.05), *p < 0.05. Baseline (*n* = 6) represents native auricles; protocol A (*n* = 3); protocol B (*n* = 3).

Young’s moduli of the auricles decellularized using protocol A did not change as compared to native auricles where the stress measurements were 2.58 and 2.75 MPa, respectively (Figure 7A). However, biomechanical testing demonstrated a decrease in Young’s moduli for auricles decellularized using protocol B (1.32 MPa), albeit not statistically significant, when compared to the native auricles and auricles decellularized using protocol A (*p* > 0.05). The DNA quantification assay demonstrated a significant drop in the mean DNA content after decellularization in both study groups (*p* < 0.05 each), where the use of protocol A and protocol B resulted in mean levels of DNA of 111.0 and 127.6 ng/mg, respectively, while the mean baseline DNA level (native auricles) was 865.3 ng/mg (Figure 7B). In addition, decellularization of auricles following protocol A methodology displayed a significant decrease in the sGAG content when compared to the amount of sGAG in native auricles (i.e., baseline sGAG of 35.1 μg/mg) (Figure 7C). The same trend observed in samples decellularized with protocol A was also noticed in auricles decellularized following protocol B methodology with the latter showing a further decrease in the sGAG content (average sGAG content was 9.3 and 4.8 μg/mg in the protocol A and protocol B study groups, respectively).

## 4 Discussion

Individuals born with microtia and outer ear anomalies are at increased risk of psychological stress during their lifetime.^2,24^ Ear anomalies could potentially be addressed with timely reconstructive procedures.^25^ However, reconstructive surgery using autologous cartilage is complex and demands advanced surgical skills.^1^ As tissue engineering strategies advance, repair of microtia has shown promise.^15,22,26^ As with any regenerated tissue, there is a trade-off among multiple elements. In the case of pinna regeneration, the trade-off is between generating a mechanically stable scaffold with high fidelity versus utilizing a material that allows for cell migration and differentiation. Although solid synthetic materials, such as alloplastics, can be used to produce a high fidelity ear-shaped construct, especially if a 3D printer is used, they are associated with an increased risk of infection, exposure, and fracture over time.^15^ Other scaffolds made from softer materials, such as hydrogel-based materials, may possess better cell conduction properties and, in certain cases, allow for the inclusion of growth factors, but they do not possess the necessary mechanical properties to be fabricated into an aesthetic outer ear.^27−29^ A biological scaffold is hypothesized to provide balanced features by retaining the original shape and contours of the ear while possessing the microstructure and growth factors that would allow for scaffold integration in the host.

This study is unique in providing a comparison of two decellularization protocols. Protocol A was developed by Ansari et al. for laryngeal decellularization and has not been reported to be used on auricle cartilage.^17^ Protocol B was adapted with modification from a study published by Rahman et al.^22^ where they compared three protocols for auricle decellularization, and they found that the addition of trypsin resulted in a significant reduction in DNA content and cell depletion. Overall, our study reproduced the reported impact of those protocols on cartilaginous tissue despite the difference in the composition of the larynx (composed mostly of hyaline cartilage) versus the auricle (composed of elastic cartilage). Ansari et al. reported a statistically significant reduction in sGAG and DNA content (below 50 ng/mg) in the decellularized laryngeal cartilage. Additionally, similar to our observation, there was no significant reduction in the tensile strength of cartilaginous specimens.^17^ Rahman et al. reported a reduction in DNA content from 55.4 to 17.3 ng/μL, and a 0.88-fold reduction of sGAG content in decellularized auricles.^22^ Rahman et al. also reported no statistically significant reduction in Young’s modulus, and their SEM study demonstrated preservation of cartilage morphology.^22^ A cutoff value of 50 ng/mg or less of DNA content per dry weight has been previously adopted to determine the effectiveness of a decellularization protocol. However, the utility of this cutoff value in a variety of tissue types is still to be validated. The desired outcome from reducing DNA content is to reduce the immunogenicity of the scaffold. Thus, an in vivo study would ultimately determine if the reduction in DNA content was effective in preventing scaffold rejection. In our study, protocol A and protocol B resulted in mean levels of DNA of 111.0 and 127.6 ng/mg, respectively. As noted above, Ansari et al. achieved reduction below 50 ng/mg in decellularized laryngeal hyaline cartilage, while Rahman et al. did not report DNA content per dry weight, but rather by volume, which is unconventional and does not allow for a comparison.^17,22^ In our study, adding an additional treatment with DNeasy could achieve a mean reduction in DNA to below 50 ng/mg. However, each treatment in a decellularization protocol is likely to affect more than one parameter. The purpose of decellularization is to achieve balanced outcomes among multiple parameters. Thus, we consider an essential next step is to test the immunogenicity of the scaffold in an animal model.

Ansari et al. and Rahman et al. protocols are conceptually similar as they employ physical (freezing, shaking, multiple washing steps), chemical (Triton X-100, sodium deoxycholate), and biological (enzymatic – DNase, RNase) decellularization methods.^17,22^ In our study, protocol A, which is based on the Ansari et al. protocol, also included stirring in a hypertonic solution (KCl) that facilitates cellular disruption. The fundamental difference between both protocols (A and B) was the use of latrunculin-B in protocol A versus trypsin in protocol B. Both of these agents were used to disrupt cellular adhesion. However, there is no previous study that compared trypsin to latrunculin-B directly. Trypsin/EDTA acts by targeting the cell–matrix adhesion and breaking down the peptide linkage, while latrunculin-B, a nonenzymatic toxin, acts by affecting actin polymerization.

In this work, the latrunculin-B-based decellularization technique (protocol A) showed promising results in creating tissue-engineered biological scaffolds from the human auricles. For example, Masson’s Trichrome and Safranin-O stains demonstrated better preservation of collagen and proteoglycans, respectively (Figures 4 and 5). Also, it was found that the arrangement of the matrix was maintained in auricles decellularized following protocol A as evidenced by scanning electron micrographs (Figure 6). Although it is important to retain sGAG for stem cell activity upon recellularization, a decrease in the sGAG content could be advantageous because it provides a greater opportunity for stem cell infiltration during recellularization.^30^ The generated auricular scaffold could also potentially be processed into a bioink and investigated for its role in 3D bioprinting to further advance the process of creating auricular cartilaginous scaffolds that could ultimately be used in reconstructing the auricle in patients with microtia.^31^

The trypsin/EDTA-based decellularization technique (protocol B), while effective, can impair extracellular matrix features. Protocol B effectively removed the cells and eliminated the genetic material (i.e., DNA content) (Figure 7B), but it did not preserve the protein content nor retain the biomechanical strength of the extracellular matrix (Figure 7A). Protocol B was reliant on trypsin/EDTA solution to effectively decellularize the auricles. This is because trypsin hydrolyzes specific peptide bonds on the cell surface and breaks down proteins, which adversely damages the structure of the extracellular matrix. EDTA that is added to trypsin to increase its activity acts by chelating the calcium and magnesium (in the extracellular matrix), which help in cell–cell adhesion. Additionally, it depletes acid-soluble proteins in the extracellular matrix.^32^ Due to the properties of the trypsin/EDTA, extracellular matrix components such as protein, calcium and magnesium are poorly preserved; thus, trypsin/EDTA ultimately damages the integrity of the extracellular matrix.

## 5 Conclusions

This study reported an effective decellularization technique of human auricular cartilage and provides a direct head-to-head comparison of two different decellularization protocols. In addition, it provides important insights into rationally designing an effective decellularization protocol for human auricles. Our study identified a suitable method for creating a tissue-engineered biological scaffold of the auricle for future application. Protocol A (latrunculin-B) produced superior outcomes as compared to protocol B (trypsin/EDTA) as evidenced by efficient depletion of the DNA content with the greater maintenance of extracellular matrix features and biomechanical characteristics. The outcomes of this study warrant proceeding with testing the biocompatibility and immunogenicity of the generated scaffold in vivo. The sought clinical application is developing an optimal implant for microtia repair. Potentially, the decellularized scaffolds could be used in their current form as an implant or utilized in combination with 3D bioprinting techniques and computerized tomography imaging in generating a higher fidelity mirror-image auricle of the nondeformed contralateral auricle in patients with unilateral microtia.

## References

[ref1] Bly R. A.; Bhrany A. D.; Murakami C. S.; Sie K. C. Microtia Reconstruction. Facial Plast Surg Clin North Am. 2016, 24 (4), 577–591. 10.1016/j.fsc.2016.06.011. 27712823 PMC5950715

[ref2] Luquetti D. V.; Heike C. L.; Hing A. V.; Cunningham M. L.; Cox T. C. Microtia: epidemiology and genetics. Am. J. Med. Genet., Part A 2012, 158A (1), 124–39. 10.1002/ajmg.a.34352. 22106030 PMC3482263

[ref3] Harris J.; Källén B.; Robert E. The epidemiology of anotia and microtia. Journal of Medical Genetics 1996, 33 (10), 809–813. 10.1136/jmg.33.10.809. 8933331 PMC1050757

[ref4] Mastroiacovo P.; Corchia C.; Botto L. D.; Lanni R.; Zampino G.; Fusco D. Epidemiology and genetics of microtia-anotia: a registry based study on over one million births. J. Med. Genet 1995, 32 (6), 453–7. 10.1136/jmg.32.6.453. 7666397 PMC1050485

[ref5] Forrester M. B.; Merz R. D. Descriptive epidemiology of anotia and microtia, Hawaii, 1986–2002. Congenital Anomalies 2005, 45 (4), 119–24. 10.1111/j.1741-4520.2005.00080.x. 16359491

[ref6] Canfield M. A.; Langlois P. H.; Nguyen L. M.; Scheuerle A. E. Epidemiologic features and clinical subgroups of anotia/microtia in Texas. Birth Defects Res., Part A 2009, 85 (11), 905–13. 10.1002/bdra.20626. 19760683

[ref7] Storck K.; Staudenmaier R.; Buchberger M.; Strenger T.; Kreutzer K.; von Bomhard A.; Stark T. Total reconstruction of the auricle: our experiences on indications and recent techniques. BioMed Res. Int. 2014, 2014, 373286 10.1155/2014/373286. 24822198 PMC4005147

[ref8] Ali K.; Meaike J. D.; Maricevich R. S.; Olshinka A. The Protruding Ear: Cosmetic and Reconstruction. Semin Plast Surg 2017, 31 (3), 152–160. 10.1055/s-0037-1604241. 28798550 PMC5550315

[ref9] Olshinka A.; Louis M.; Truong T. A. Autologous Ear Reconstruction. Semin Plast Surg 2017, 31 (3), 146–151. 10.1055/s-0037-1603959. 28798549 PMC5550308

[ref10] Firmin F.; Marchac A. A novel algorithm for autologous ear reconstruction. Semin Plast Surg 2011, 25 (4), 257–64. 10.1055/s-0031-1288917. 23115531 PMC3312152

[ref11] Sterodimas A.; de Faria J.; Correa W. E.; Pitanguy I. Tissue engineering and auricular reconstruction: a review. J. Plast Reconstr Aesthet Surg 2009, 62 (4), 447–52. 10.1016/j.bjps.2008.11.046. 19114322

[ref12] Baiguera S.; Gonfiotti A.; Jaus M.; Comin C. E.; Paglierani M.; Del Gaudio C.; Bianco A.; Ribatti D.; Macchiarini P. Development of bioengineered human larynx. Biomaterials 2011, 32 (19), 4433–42. 10.1016/j.biomaterials.2011.02.055. 21474177

[ref13] Dzobo K.; Thomford N. E.; Senthebane D. A.; Shipanga H.; Rowe A.; Dandara C.; Pillay M.; Motaung K. Advances in Regenerative Medicine and Tissue Engineering: Innovation and Transformation of Medicine. Stem Cells Int. 2018, 2018, 2495848 10.1155/2018/2495848. 30154861 PMC6091336

[ref14] Parveen S.; Krishnakumar K.; Sahoo S. New era in health care: tissue engineering. J. Stem Cells Regen Med. 2006, 1, 8–24. 10.46582/jsrm.0101003. 24692857 PMC3907955

[ref15] Reighard C. L.; Hollister S. J.; Zopf D. A. Auricular reconstruction from rib to 3D printing. J. 3D Print Med. 2018, 2 (1), 35–41. 10.2217/3dp-2017-0017. 29607095 PMC5824712

[ref16] Otto I. A.; Capendale P. E.; Garcia J. P.; de Ruijter M.; van Doremalen R. F. M.; Castilho M.; Lawson T.; Grinstaff M. W.; Breugem C. C.; Kon M.; Levato R.; Malda J. Biofabrication of a shape-stable auricular structure for the reconstruction of ear deformities. Materials Today Bio 2021, 9, 100094 10.1016/j.mtbio.2021.100094. PMC7903133 33665603

[ref17] Ansari T.; Lange P.; Southgate A.; Greco K.; Carvalho C.; Partington L.; Bullock A.; MacNeil S.; Lowdell M. W.; Sibbons P. D.; Birchall M. A. Stem Cell-Based Tissue-Engineered Laryngeal Replacement. Stem Cells Transl. Med. 2017, 6 (2), 677–687. 10.5966/sctm.2016-0130. 28191770 PMC5442815

[ref18] Londono R.; Badylak S. F. Biologic scaffolds for regenerative medicine: mechanisms of in vivo remodeling. Ann. Biomed. Eng. 2015, 43 (3), 577–92. 10.1007/s10439-014-1103-8. 25213186

[ref19] Costa A.; Naranjo J. D.; Londono R.; Badylak S. F. Biologic Scaffolds. Cold Spring Harbor Perspect. Med. 2017, 7 (9), 1–23. 10.1101/cshperspect.a025676. PMC5580515 28320826

[ref20] Li Y.; Zhu T.; Wang L.; Jiang J.; Xie G.; Huangfu X.; Dong S.; Zhao J. Tissue-Engineered Decellularized Allografts for Anterior Cruciate Ligament Reconstruction. ACS Biomater. Sci. Eng. 2020, 6 (10), 5700–5710. 10.1021/acsbiomaterials.0c00269. 33320573

[ref21] Al-Qurayshi Z.; Wafa E. I.; Hoffman H.; Chang K.; Salem A. K. Tissue-engineering the larynx: Effect of decellularization on human laryngeal framework and the cricoarytenoid joint. J. Biomed. Mater. Res., Part B 2021, 1–11. 10.1002/jbm.b.34851. PMC9668637 33872461

[ref22] Rahman S.; Griffin M.; Naik A.; Szarko M.; Butler P. E. M. Optimising the decellularization of human elastic cartilage with trypsin for future use in ear reconstruction. Sci. Rep. 2018, 8 (1), 3097 10.1038/s41598-018-20592-x. 29449572 PMC5814427

[ref23] Uysal O.; Arslan E.; Gulseren G.; Kilinc M. C.; Dogan I.; Ozalp H.; Caglar Y. S.; Guler M. O.; Tekinay A. B. Collagen Peptide Presenting Nanofibrous Scaffold for Intervertebral Disc Regeneration. ACS Applied Bio Materials 2019, 2 (4), 1686–1695. 10.1021/acsabm.9b00062. 35026903

[ref24] Hamlet C.; Harcourt D. Exploring the Experiences of Adults With Microtia: A Qualitative Study. Cleft Palate Craniofac J. 2020, 57 (10), 1230–1237. 10.1177/1055665620931611. 32643387 PMC7502977

[ref25] Johns A. L.; Lucash R. E.; Im D. D.; Lewin S. L. Pre and post-operative psychological functioning in younger and older children with microtia. J. Plast Reconstr Aesthet Surg 2015, 68 (4), 492–7. 10.1016/j.bjps.2014.12.019. 25573811

[ref26] Cohen B. P.; Bernstein J. L.; Morrison K. A.; Spector J. A.; Bonassar L. J. Tissue engineering the human auricle by auricular chondrocyte-mesenchymal stem cell co-implantation. PLoS One 2018, 13 (10), e0202356 10.1371/journal.pone.0202356. 30356228 PMC6200177

[ref27] El-Sherbiny I. M.; Yacoub M. H. Hydrogel scaffolds for tissue engineering: Progress and challenges. Glob Cardiol Sci. Pract 2013, 2013 (3), 316–42. 10.5339/gcsp.2013.38. 24689032 PMC3963751

[ref28] Nikolova M. P.; Chavali M. S. Recent advances in biomaterials for 3D scaffolds: A review. Bioact Mater. 2019, 4, 271–292. 10.1016/j.bioactmat.2019.10.005. 31709311 PMC6829098

[ref29] Shin J.; Kang E. H.; Choi S.; Jeon E. J.; Cho J. H.; Kang D.; Lee H.; Yun I. S.; Cho S. W. Tissue-Adhesive Chondroitin Sulfate Hydrogel for Cartilage Reconstruction. ACS Biomater. Sci. Eng. 2021, 10.1021/acsbiomaterials.0c01414 33538598

[ref30] Schwarz S.; Koerber L.; Elsaesser A. F.; Goldberg-Bockhorn E.; Seitz A. M.; Durselen L.; Ignatius A.; Walther P.; Breiter R.; Rotter N. Decellularized cartilage matrix as a novel biomatrix for cartilage tissue-engineering applications. Tissue Eng., Part A 2012, 18 (21–22), 2195–209. 10.1089/ten.tea.2011.0705. 22690787

[ref31] Apelgren P.; Karabulut E.; Amoroso M.; Mantas A.; Martínez Ávila H.; Kölby L.; Kondo T.; Toriz G.; Gatenholm P. In Vivo Human Cartilage Formation in Three-Dimensional Bioprinted Constructs with a Novel Bacterial Nanocellulose Bioink. ACS Biomater. Sci. Eng. 2019, 5 (5), 2482–2490. 10.1021/acsbiomaterials.9b00157. 33405755

[ref32] Schenke-Layland K.; Vasilevski O.; Opitz F.; Konig K.; Riemann I.; Halbhuber K. J.; Wahlers T.; Stock U. A. Impact of decellularization of xenogeneic tissue on extracellular matrix integrity for tissue engineering of heart valves. J. Struct. Biol. 2003, 143 (3), 201–8. 10.1016/j.jsb.2003.08.002. 14572475

